# Metastatin as a Marker for Hyperandrogenemia in Iraqi Women with Polycystic Ovary Syndrome

**DOI:** 10.1155/2020/5216903

**Published:** 2020-09-25

**Authors:** Manal Madany Abdalqader, Shatha Sami Hussein

**Affiliations:** College of Medicine, Department of Obstetrics &Gynecology, Al-Mustansiriyah University, Baghdad, Iraq

## Abstract

**Objective:**

Kisspeptin 1 might reflect increased androgen level in polycystic ovarian syndrome instead of other markers. *Study Design*. A case control study was performed in Al-Yarmouk Teaching Hospital from 1^st^ of July 2016 to 1^st^ of July 2017; it involved 87 women divided into two groups: 44 women diagnosed as PCOS, 22 women with BMI ≥ 25 kg/m^2^ and 22 women with BMI < 25 kg/m^2^, and another 43 women without PCOS, 22 women with BMI ≥ 25 kg/m^2^ and 21 women with BMI < 25 kg/m^2^. Hormonal, metabolic profiles, and hirsutism scores, as well as serum kisspeptin level, were assessed by using Human Kisspeptin 1(KISS-1) ELISA Kit. The blood samples between days 2 and 5 of menstrual cycle were drawn by an disposable sterile syringe and collected in EDTA containing tubes (as anticoagulant), and the hormonal profile was measured using a biotech ELISA reader.

**Result:**

Serum level of kisspeptin was significantly higher in PCOS compared to control (322.4 vs. 235.3 ng/L, respectively). There was no significant difference in age, BMI, and parity between control and PCOS; the frequency of hirsutism, acne, elevated LH, and increased free testosterone (fTT) were significantly higher in PCOS compared to control. Kisspeptin shows a direct significant correlation with hirsutism and fTT (*r* = 0.648, 0.238, respectively). In ROC analysis, kisspeptin had AUC (95% CI) = 0.874 (0.785–0.935) for predicting PCOS.

**Conclusion:**

Kisspeptin levels might be used as a marker for hyperandrogenemia in polycystic ovarian syndrome.

## 1. Introduction

Polycystic ovarian syndrome (PCOS) is regarded as one of the commonest endocrinopathic disorders in women at the reproductive age group with a prevalence of about 6–12% [1]. It is usually characterized by multiple hormonal imbalances, reflecting as a sign and symptom of hyperandrogenism, which leads to multiple health effects on females in the form of early and late sequelae [2], with androgen excess, oligomenorrhea or an ovulation, and ultrasound finding of polycystic ovary appearance. In addition, other abnormalities such as insulin resistance, inflammation, obesity, cardiovascular changes, and infertility are common among women with PCOS [3]. Although up to 70% of women with PCOS may be undiagnosed and due to high prevalence of PCOS [4], all cases of hirsutism during puberty or in women of reproductive age group merit investigations for sex hormone level, pelvic ultrasonography, to exclude this syndrome [5].

A pivotal role for specific proinflammatory mediators in the pathogenesis of PCOS was described in recent evidence which elucidated a new pathway on the etiological considerations for PCOS, as it recently considered a chronic, low-grade inflammatory abnormality, independent for the presence of obesity [6]. Kisspeptin is a gene product, which usually resulted from an initial 145-amino acid peptide which cleaved to a 54-amino acid protein [7]. Kisspeptin has a role in gonadotropin-releasing hormone (GnRH) neuron activation in the hypothalamus, and as a result, it causes GnRH release which leads to FSH and LH release. Kisspeptin/GPR54 plays a major role in the initial sexual development which is found in mice and sexually immature humans with mutations that block the expression of the GPR54 gene [8].

Several independent lab groups support the role of kisspeptin in the hypogonadotropic hypogonadism pathway. GPR54 mutation was found to be the reason for this abnormality, as it was noted that people who had this mutation, or missing GPR54, have abnormality in the development of gonads at puberty. Other form of phenotypes was connected to this mutation including gonadotropin concentrations, a low level of sex steroid in blood, and sterility which led to discover the role of Kisspeptin in initiation of puberty by triggering the neurons which participate in GnRH release and the possible role on of luteinizing hormone (LH) and follicle-stimulating hormone (FSH) release [9]. Efforts were made to understand the regulatory mechanism of kisspeptin and its gene expression and more exactly detect the mechanism behind its role in GnRH and LH release.

Because of the complicated relation seen between kisspeptin and the hypothalamic-pituitary-gonadal axis, the current work planned to measure the level of kisspeptin in polycystic ovary syndrome (PCOS) and analyze the relation between kisspeptin and PCOS-related reproductive and associated metabolic changes.

## 2. Materials and Methods

### 2.1. Study Design

A case control study was carried out in Obstetric and Gynecological Department at Al-Yarmouk Teaching Hospital through the period from 1^st^ of July 2016 to 1^st^ of July 2017. A total of 87 women participated in this study and were divided into 2 groups: group A: 44 women diagnosed as PCOS (divided into 22 women with BMI ≥ 25 kg/m^2^ and 22 women with BMI < 25 kg/m^2^) and group B: 43 women without PCOS (divided into 22 women with BMI ≥ 25 kg/m^2^ and 21 women with BMI < 25 kg/m^2^).

### 2.2. Inclusion and Exclusion Criteria

Inclusion criteria included women diagnosed as PCOS according to the Rotterdam criteria [10] in the form of oligomenorrhea ± an ovulation, ultrasound of polycystic ovaries (follicular cyst number  ≥ 12), and biochemical and/or clinical evidence of hyperandrogenism. Exclusion criteria included any cause of hyperandrogenism other than PCOS as congenital adrenal hyperplasia, virilization tumors, Cushing syndrome, prolactinoma, hypertension, diabetes, and cardiovascular disorders.

### 2.3. Data Collection

Full history regarding signs and symptoms (hirsutism, acne, menstrual irregularity, amenorrhea etc.) and examination which includes BMI, hirsutism evaluation according to modified Ferriman–Gallwey score ≥8 [11] was collected; calculation of their BMI included the ratio of weight divided by the square of height (kg/m^2^). Routine laboratory investigation was performed including fasting plasma glucose (FPG), HDL, LDL, triglyceride, LH, FSH, TSH, sex hormone-binding globulin (SHBG), dehydroepiandrosterone sulfate (DHEAS), and testosterone level which were all taken at the morning after sample for serum overnight fasting at the early follicular phase (2^nd^–5^th^ day of the menstrual cycle). Kisspeptin levels were also taken and performed by an enzyme immunoassay kit. Based on the findings of Gorkem et al. [12] with mean difference of mean (1.11) and standard deviation (SD) for PCOS (2.11) and SD for control (2.16) for kisspeptin, with type I error (*α*) = 0.10 and type II error (*β*) = 80%, the sample size was estimated to be 47 for PCOS and 47 for control.

### 2.4. Flowchart

After initial selection of the groups, 8.5% (4 women) of subjects in PCOS and 6.3% (3 women) in the control group were excluded from the study. Since both were less than 20% (which is considered significant bias), the authors did not consider this lost to follow-up because it would create significant bias [13].

### 2.5. Statistical Analysis

**Figure 1:**
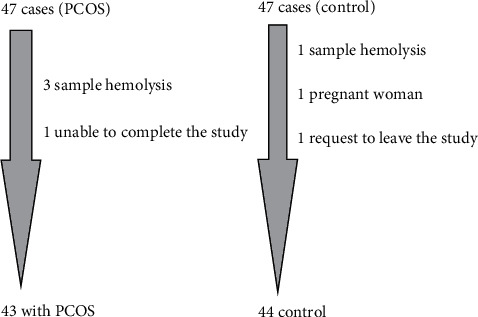
Flowchart of the study.

The age was presented as mean and standard deviation as it was normally distributed, while both parity and kisspeptin were presented as median and interquartile range (did not follow normal Mann distribution, assessed by Kolmogorov–Smirnov test for continuous data distribution). Whitney U nonparametric test (two-tailed) was used to compare the continuous variables; receiver operating characteristic (ROC) curve was used to assess the sensitivity between study groups. Pearson's chi-square test was used to assess specificity of kisspeptin of participants in both groups and to assess statistical association between the categorical parameter levels and the study groups. Linear regression analysis was used to assess the relationship between kisspeptin and other parameters. ROC analysis was performed to assess the diagnostic validity of kisspeptin for PCOS. A *p* value less than 0.05 was significant, and all the statistical analyses were performed using SPSS 22.0.0 (Chicago, IL) and MedCalc Statistical Software version 14.8.1 (MedCalc Software bvba, Ostend, Belgium; 2014) (Figure 1).

### 2.6. Results

**Figure 2:**
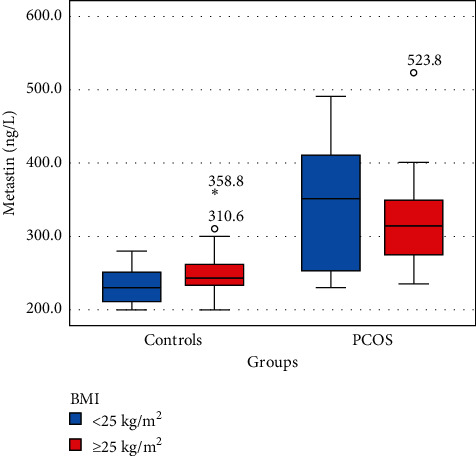
Plasma kisspeptin (metastin) levels of women with regard to the body mass index (asterisk and small circles represent the outliers).

There was no significant difference in age, BMI, and parity between control and PCOS; the frequency of hirsutism, acne, elevated LH, and increased free testosterone (fTT) were significantly higher in PCOS compared to control, and serum levels of kisspeptin were also significantly higher in PCOS (this maintained even after dividing both groups according to their BMI, see Figure 2), as illustrated in Table 1.

Kisspeptin shows a direct significant correlation with hirsutism and fTT as illustrated in Table 2.

**Figure 3:**
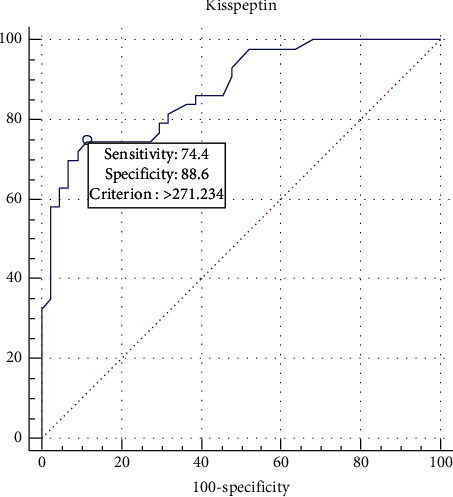
ROC analysis for kisspeptin levels between the PCOS and the control groups.

Kisspeptin shows good ability (since AUC between 0.8 and 0.89) to predict PCOS, since SP (specificity) is higher than SN (sensitivity), and also PPV (positive predictive value) is higher than NPV (negative predictive value); this suggests it may be a more useful tool for confirmation of the diagnosis of PCOS, as illustrated in Table 3 and Figure 3.

In multivariate analysis, kisspeptin shows an independent relationship with PCOS (after excluding the effect possible confounders in this study such as age, BMI, LH, and fTT) in which an increase in 1 ng/L of kisspeptin increased the probability of PCOS by 3.9% (ranging from 1.9% to 5.9%; ARR: 1.039–1.0), as illustrated in Table 4.

## 3. Discussion

PCOS is related to a disturbance in the hypothalamic-pituitary-gonadal axis [14]. Kisspeptin has been recognized by its important role in GnRH secretion initiation at puberty. Kisspeptin has been recognized by its important role in GnRH secretion initiation at puberty, regulatory secretion of LH during ovulation process, and its relation to PCOS was found to be implicated in the hypothalamus-pituitary-ovary axis disturbance observed in polycystic ovary syndrome (PCOS) [15].

The current study showed that kisspeptin was significantly higher in PCOS compared to control (322.4 vs. 235.3 ng/L), and after performing ROC analysis, kisspeptin showed good ability to predict PCOS, with serum level ≥271.234 ng/L predicting PCOS; at this point, kisspeptin showed higher specificity (88.6%) and lower sensitivity (74.4%) with 81.6% accuracy. In addition, the positive likelihood ratio (LH) was 6.55 which indicates that this test can increase the index of suspicion for confirming the PCOS diagnosis by 35–40%, while its negative LH (0.29) indicates it has 25–30% ability to exclude the diagnosis of PCOS; this was in agreement with a recent Turkish study in 2018 with kisspeptin levels in a normal ovarian reserve which were 4.65 ± 2.16 ng/mL, and 5.76 ± 2.11 in those with a high ovarian reserve [12]. Jeon et al. showed similar findings (10.65 ± 6.14 vs. 6.51 ± 3.13 pmol/l, *p* < 0.001) [16] and were in agreement with Umayal et al. (4.713 vs. 4.127 nmol/L, *p* value = 0.033) [17]. Other studies such as Chen et al. and Joen et al. both showed increased kisspeptin level in women with PCOS [16, 18]. Others showed no significant difference between PCOS and control, such as Ozay et al. [19] with kisspeptin levels 1.92 ± 1.29 vs. 1.49 ± 1.46; *p* value = 0.638 [19], all these indicate that kisspeptin had good potential as a marker for PCOS diagnosis.

Some studies showed that kisspeptins may play a role in the regulation of islet function by stimulating insulin secretion from mouse and human islets. Adipose tissue acts as an endocrine organ, and its function may be regulated by kisspeptins [20]. It has been shown that peptides have more gonadotropin secreted through the activation of the central hypothalamic-pituitary-gonadal (HPG) axis at the hypothalamic level, with the gonadotropin stimulatory effects of kisspeptin being abolished by preadministration of a GnRH antagonist [21]; therefore, kisspeptin was found to have a regulatory effect through the activation of GnRH neurons, making it the first step for the last common pathway associated with controlling reproduction.

Increased HPG axis stimulation may be associated with reproductive function restoration for PCOS women, while overstimulation of this pathway axis produces downregulation of reproductive hormone secretion, and KISS-1 is found to have a role in treating prostate and breast malignancies, as well as benign hormone-dependent disorders as benign prostate hyperplasia and endometriosis, through its act as a suppresser in the metastatic cascade [22].

## 4. Conclusion

In the current study, the effects of kisspeptin on the release of reproductive hormones were examined. All the above findings have identified kisspeptin as a possible diagnostic marker for PCOS.

## Figures and Tables

**Table 1:** Comparison between control and PCOS women.

Parameters	Control	PCOS	*p* value
Number	44	43	—
Age (years), mean ± SD	24.5 ± 5.6	26.3 ± 6.0	0.162
Parity, median (IQR)	1 (0.25–2.0)	2 (1–3)	0.120
Kisspeptin (ng/L), median (IQR)	235.3 (220.6–258.4)	322.4 (254.6–377.1)	<0.001
BMI, *n* (%)			
<25 kg/m^2^	21 (47.7%)	23 (53.5%)	0.591
≥25 kg/m^2^	23 (52.3%)	20 (46.5%)	
Hirsutism, *n* (%)	0 (0%)	43 (100%)	<0.001
Acne, *n* (%)	18 (40.9%)	27 (62.8%)	0.041
Elevated LH, *n* (%)	14 (31.8%)	27 (62.8%)	0.004
FSH, *n* (%)			
Normal	31 (70.5%)	28 (65.1%)	0.700
Decreased	9 (20.5%)	12 (27.9%)	
Elevated	4 (9.1%)	3 (7%)	
Increased TSH, *n* (%)	11 (25%)	13 (30.2%)	0.585
Increased PRL, *n* (%)	20 (45.5%)	15 (34.9%)	0.315
SHBG, *n* (%)			
Normal	31 (70.5%)	29 (67.4%)	0.952
Decreased	11 (25%)	12 (27.9%)	
Elevated	2 (4.5%)	2 (4.7%)	
Increased testosterone, *n* (%)	18 (40.9%)	28 (65.1%)	0.024
DHEAS, *n* (%)			
Normal	31 (70.5%)	30 (69.8%)	0.923
Decreased	8 (18.2%)	7 (16.3%)	
Elevated	5 (11.4%)	6 (14%)	
Glycemic status, *n* (%)			
Normal	25 (56.8%)	21 (48.8%)	0.695
Decreased	9 (20.5%)	9 (20.9%)	
Elevated	10 (22.7%)	13 (30.2%)	
Impaired lipid profile	14 (31.8%)	16 (37.2%)	0.597

SD: standard deviation, *n*: number, IQR: interquartile range (25^th^–57^th^ percentile), PRL: prolactin, SHBG: sex hormone-binding globulin, T: testosterone, DHEAS: dehydroepiandrosterone sulfate, and FPG: fasting blood glucose.

**Table 2:** Correlations between kisspeptin with demographic and clinical characteristics and hormonal and metabolic profiles of all subjects (including controls).

Parameters	Correlation coefficient (*r*)	*p* value
Age	0.016	0.886
Parity	0.131	0.228
BMI	0.066	0.541
Hirsutism	0.648	<0.001^*∗*^
Acne	0.073	0.500
LH	0.139	0.199
FSH	−0.194	0.072
TSH	−0.045	0.679
PRL	−0.133	0.218
SHBG	−0.032	0.772
Testosterone	0.238	0.027^*∗*^
DHEAS	−0.101	0.353
FPG	−0.064	0.558

PRL: prolactin, SHBG: sex hormone-binding globulin, DHEAS: dehydroepiandrosterone sulfate, and FPG: fasting blood glucose.

**Table 3:** ROC analysis of the validity of kisspeptin as a predictor of PCOS.

ROC (95% CI)	Cutoff	SN	SP	AC (%)	PPV	NPV	+LH	−LH
0.874 (0.785–0.935)	≥271.234	74.4	88.6	81.6	86.5	78.0	6.55	0.29

SN: sensitivity, SP: specificity, AC: accuracy, PPV: positive predictive value, NPV: negative predictive value, and LH: likelihood ratio.

**Table 4:** Multiple regression analysis for the *e* factors that affect PCOS.

Parameters	OR (95% CI)	Wald	*p* value
Age	1.106 (0.987–1.240)	2.999	0.083
BMI	0.440 (0.110–1.755)	1.353	0.245
Kisspeptin	1.039 (1.019–1.059)	15.313	>0.001 [S]
LH	12.002 (2.543–56.651)	9.852	0.002 [S]
Free TT	3.385 (0.870–3.179)	3.092	0.079

*R* ^2^ (Cox and Snell) = 0.514.

## Data Availability

The patient data used to support the findings of this study are currently under embargo, while the research findings are commercialized. Requests for data 6/12 months after publication of this article will be considered by the corresponding author.
